# Effect of transcranial direct current stimulation on the psychomotor, cognitive, and motor performances of power athletes

**DOI:** 10.1038/s41598-021-89159-7

**Published:** 2021-05-06

**Authors:** Sidney Grosprêtre, Yohan Grandperrin, Magali Nicolier, Philippe Gimenez, Chrystelle Vidal, Gregory Tio, Emmanuel Haffen, Djamila Bennabi

**Affiliations:** 1 EA4660, C3S Laboratory, C3S Culture Sport Health Society, Université de Bourgogne Franche-Comté, UPFR Sports, 31, Chemin de l’Epitaphe, 25000 Besançon, France; 2 Service de Psychiatrie de L’Adulte, Centre Hospitalier Universitaire de Besançon, 25030 Besançon Cedex, France; 3 Centre D’Investigation Clinique, INSERM CIC 1431, Centre Hospitalier Universitaire de Besançon, 25030 Besançon Cedex, France; 4 Laboratoire de Neurosciences Intégratives et Cliniques EA481, Université de Bourgogne Franche-Comté, 19 rue Ambroise Paré, 25000 Besançon, France; 5 Centre Expert Dépression Résistante FondaMental, Centre Hospitalier Universitaire de Besançon, 25030 Besançon Cedex, France

**Keywords:** Cognitive neuroscience, Neurophysiology

## Abstract

In sports science, transcranial direct current stimulation (tDCS) has many unknown effects on neuromuscular, psychomotor and cognitive aspects. Particularly, its impact on power performances remains poorly investigated. Eighteen healthy young males, all trained in a jumping sport (parkour) performed three experimental sessions: anodal tDCS applied either on the left dorsolateral prefrontal cortex (dlPFC, cathode in supraorbital area) or on the primary motor cortex (M1, cathode on contralateral shoulder), and a placebo condition (SHAM), each applied for 20 min at 2 mA. Pre and post, maximal vertical and horizontal jumps were performed, associated to leg neuromuscular assessment through electromyography and peripheral nerve stimulations. Actual and imagined pointing tasks were also performed to evaluate fine motor skills, and a full battery of cognitive and psychomotor tests was administered. M1 tDCS improved jump performance accompanied by an increase in supraspinal and spinal excitabilities. dlPFC stimulation only impacted the pointing tasks. No effect on cognitive tests was found for any of the tDCS conditions. To conclude, the type of performance (maximal versus accurate) affected depended upon the tDCS montage. Finally, athletes responded well to tDCS for motor performance while results to cognitive tests seemed unaffected, at least when implemented with the present rationale.

## Introduction

In the twenty-first century, new training techniques inspired by advances in the neurosciences have been developed. Among them, the use of non-invasive brain stimulation, initially developed for clinical purposes, has expanded to the field of sports performance. The most common technique, transcranial direct current stimulation (tDCS), has increased in popularity due to its safety, low-cost, and ease of implementation. tDCS consists of applying a constant low intensity current (1 to 2 mA) on targeted brain areas for several minutes (10 to 30 min), which can induce changes in cortical excitability lasting up to 5 h^1^. For a decade, many studies investigated the effect of tDCS on physical performance with a wide range of rationales, from tDCS settings to tested exercises^2^. In this regard, the literature has mainly focused on endurance performance involving either a single joint^3–5^ or whole-body exercises^6,7^ by increasing the potential effect of brain stimulation over the perception of effort^4–6,8^. Studies have shown that tDCS enhances neural factors, such as motor unit synchronization^9,10^ or motor cortex excitability^11^, which are implied also in an explosive effort, i.e., the ability to produce a maximal force in a short amount of time. However, very few studies have investigated the effects of tDCS on this type of performance, and when it was addressed, divergent results were observed. For example, Lattari et al. found an increase in vertical jump performance^12^, while two other studies did not^13,14^. These discrepancies might be due to difference in the study’s rationales, in terms of the targeted brain area (motor cortex versus dorsolateral prefrontal cortex) or the tested population (trained versus untrained). Here, we propose a single study to understand the effect of tDCS on explosive performance according to those different parameters.

First, we aim to provide new insights by targeting two brain areas frequently assessed in the literature: the primary motor (M1) and left dorsolateral prefrontal (dlPFC) cortices. While the role of M1 is to elaborate the motor program and, via the pyramidal tract, activate the spinal motoneurons, dlPFC function is more related to motor planning, attention, or working memory^15,16^. The literature about tDCS and power-performance, although weak in this area, is not equivocal since to alter jump performances some authors targeted either the dlPFC^13^ or M1^12^. In a general manner, addressing the question whether motor or cognitive skills are modulated by stimulating specific regions is of interest, while most of the literature assessed one single area. This would help understanding if actively stimulating a non-specific region could lead to a transfer of effect. It should also be mentioned that the type of tDCS used here being not focal the effects could be diffuse and affect adjacent brain areas, therefore still inducing modulation of cognitive and/or motor functions.

Second, we propose to assess the effect of tDCS on athletes with different levels of expertise, ranging from a few training hours up to 10,000 h. Particularly, parkour, which consists of jumping over various obstacles mostly in an urban landscape, has been recognized as one of the most power-typed sport activities^17^. Athletes practicing parkour present a specific neuromuscular profile^18^ that could make them particularly prone to responding to tDCS interventions. Furthermore, they are used to practicing the jumping tasks they will be tested for, eluding a possible learning effect across the protocol.

The present study proposes to cover a wide range of brain and neuromuscular functions, from higher centers that manage high cognitive processes to lower nervous networks that control the recruitment of motor units. To unveil the effects of tDCS on these different levels, a full battery of cognitive tests, motor tasks, and neuromuscular evaluations were used to investigate the multiple factors that can contribute to a better sports performance. The study’s protocol, methods and materials were fully described and detailed in a specific article that has been previously published^19^.

**Figure 1:**
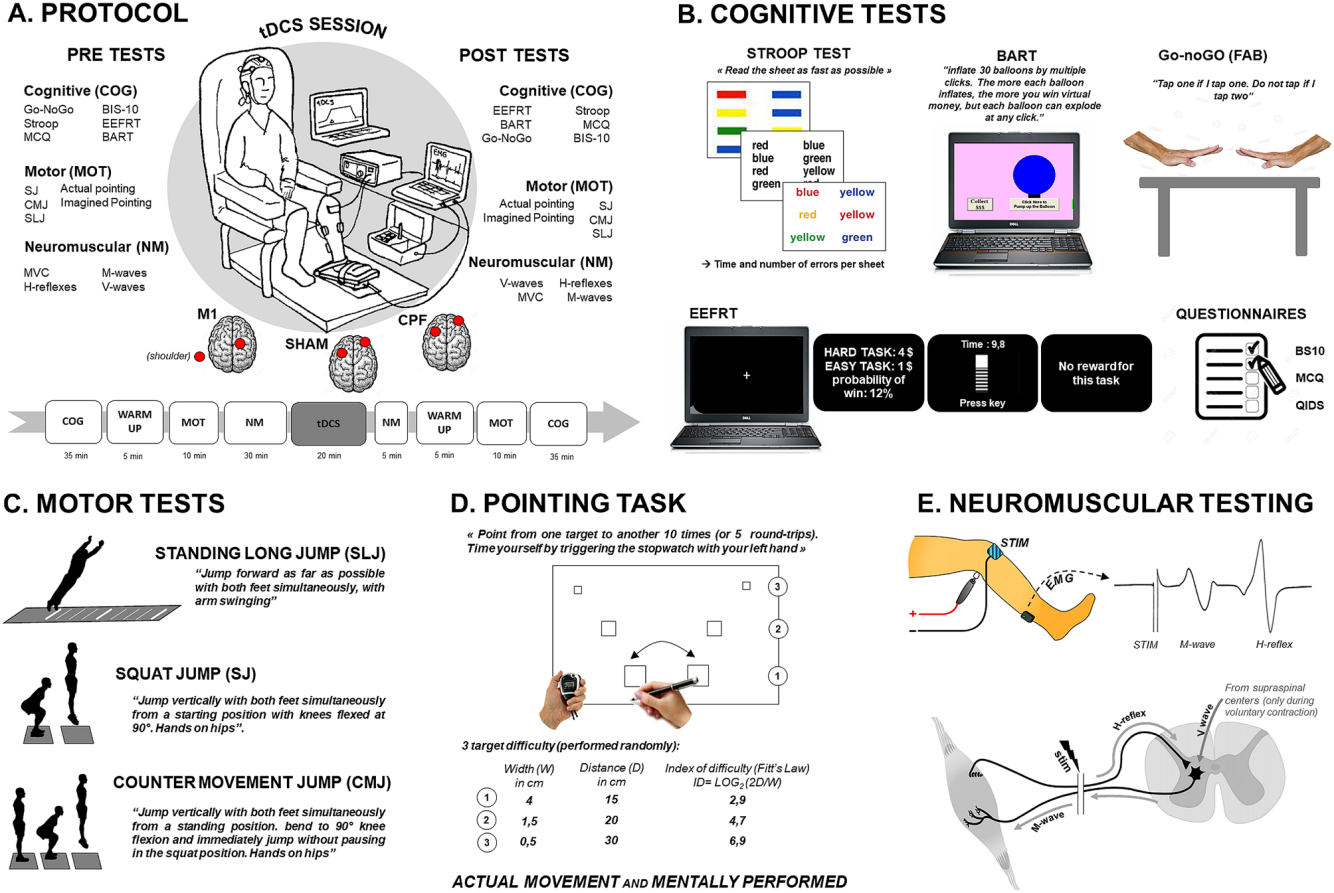
Overview of the experimental protocol. **(A)** General picture of the experimental protocol with each pre- and post-measurement detailed in the other panels. The three tDCS conditions are displayed under the illustration of the experimental setup, representing each of the three sessions: (1) anode over the right M1 area and cathode over the left shoulder; (2) anode over the left dlPFC and cathode over the right supra-orbital area; (3) placebo condition (SHAM with same experimental setup as used for the second session (dlPFC). A timeline of the experimental sessions is also depicted. (**B)** Cognitive tasks: Stroop test, Balloon Analog Risk Task (BART), Go/No-go test from the Frontal Assessment Battery (FAB), Effort Expenditure for Rewards Task (EEfRT), Monetary Choice Questionnaire (MCQ), Barratt Impulsiveness Scale (BIS-10), and Quick Inventory of Depressive Symptomatology (QIDS). (**C)** Pictures of the three different types of jump performances that were assessed. (**D)** Pointing task reflecting the management of the speed accuracy trade-off (Fitt’s law). Each level of difficulty was performed under actual conditions and mentally. (**E)** Picture depicting the principle of the neuromuscular assessment performed in the present study: the percutaneous stimulation of a mixed nerve evokes several potentials, recorded at the muscle level by electromyography. Three responses were analyzed and used to represent three neuromuscular levels: the M-wave (muscle level), resulting from the direct activation of the muscle by a depolarization of motor axons in the nerve branch; the H-reflex (spinal level), resulting from an activation of the muscle through a myotatic reflex arc; the V-wave (supraspinal level), resulting from a collision between the antidromic current from the stimulation and the voluntary descending neural drive.

To summarize, after inclusion held by a trained psychiatrist, power-athletes were recruited and asked to participate in three experimental sessions, aiming at investigating different tDCS arrangements: (1) the anode placed over the right M1 (corresponding to FC2 according to the international 10–20 EEG system) and the cathode placed over the left shoulder (M1 condition); (2) the anode placed over the left dlPFC and the cathode placed over the right supraorbital region, corresponding to F3 and AF8 according to the international 10–20 EEG system (dlPFC condition); and (3) placebo stimulation (SHAM condition) with the same montage as the dlPFC condition. In each of these sessions, tDCS was applied offline for 20 min at 2 mA, with PRE and POST evaluations (full protocol is depicted in Fig. 1A–E). The main outcomes, related to the specificity of the population tested i.e. power-athletes, were vertical jump performances in Counter Movement Jump (CMJ) and Squat Jump (SJ), and maximal horizontal jump distance (Standing Long Jump, SLJ). To account for tDCS effects on the neuromuscular system that could potentially explain a change in performance, neuromuscular function of leg muscles (triceps surae) was assessed by recording several electromyographic motor potentials evoked by peripheral nerves stimulations, recorded at rest and during maximal voluntary contraction (MVC). Submaximal and maximal H-reflexes accounted for spinal excitability changes, V-waves for supraspinal plasticity and M-waves for muscle excitability changes.

It should be noticed that parkour athletes, contrary to other power-athletes such as track and field jumpers who are in search of the maximal performance only, do have to manage a certain accuracy of their performance due to environmental constraints^20^. Performance in parkour is then not only related to the capacity of the neuromuscular system to produce the highest power as possible. Therefore, finer motor skills were also assessed by means of recording the time to perform an accurate pointing task following the Fitt’s paradigm. These pointing tasks were performed both in actual execution and mentally simulated through motor imagery to account for changes occurring in motor planning only^21^.

The aim of the present study is also to question other variables that could be modulated by tDCS which could potentially alter motor performances. Indeed, studying the effect of a given intervention on sport performance is complex, particularly with open-skilled activities such as Parkour. Consequently, only a holistic approach allows to depict a clearer picture of the mechanisms involved. Then, the cognitive and psychomotor aspects were screened through a battery of psychometric tasks that explores depressive symptomatology, impulsivity, or motivation. Participants performed computer-based tests such as the Effort Expenditure for Reward Task (EEfRT)^22^ or the Balloon Analog Risk Task (BART)^23^, psychometric tests such as Go/No-go^24^ and Stroop tasks^25^, and self-administered questionnaires such as the 27-item Monetary Choice Questionnaire (MCQ)^26^.

Since fine motor tasks involve the management of speed and accuracy and power tasks, i.e. jumps, have a higher muscular demand, the expected effects of tDCS could largely depend upon the targeted stimulated brain area. As well, cognitive and motor functions are not managed either by similar cortical circuits. Given their respective role in motor planning and execution, we hypothesized that cognitive and fine motor skills would more be affected after dlPFC stimulations, while maximal force or vertical jumps would be more likely influenced by tDCS applied over M1^27^. As well, it can be hypothesized that M1 stimulation would provide positive effect on neuromuscular function, since it was previously shown that tDCS applied over this area lead to an increase of corticospinal excitability^28^, and to a modulation of spinal circuitry^29^.

## Results

No difference was found in any parameter regarding baseline measurements (differences between the PRE data of each session). Similarly, no session-order effect has been statistically identified when analyzing data chronologically, regardless of the condition.

### Jump performances

**Figure 2:**
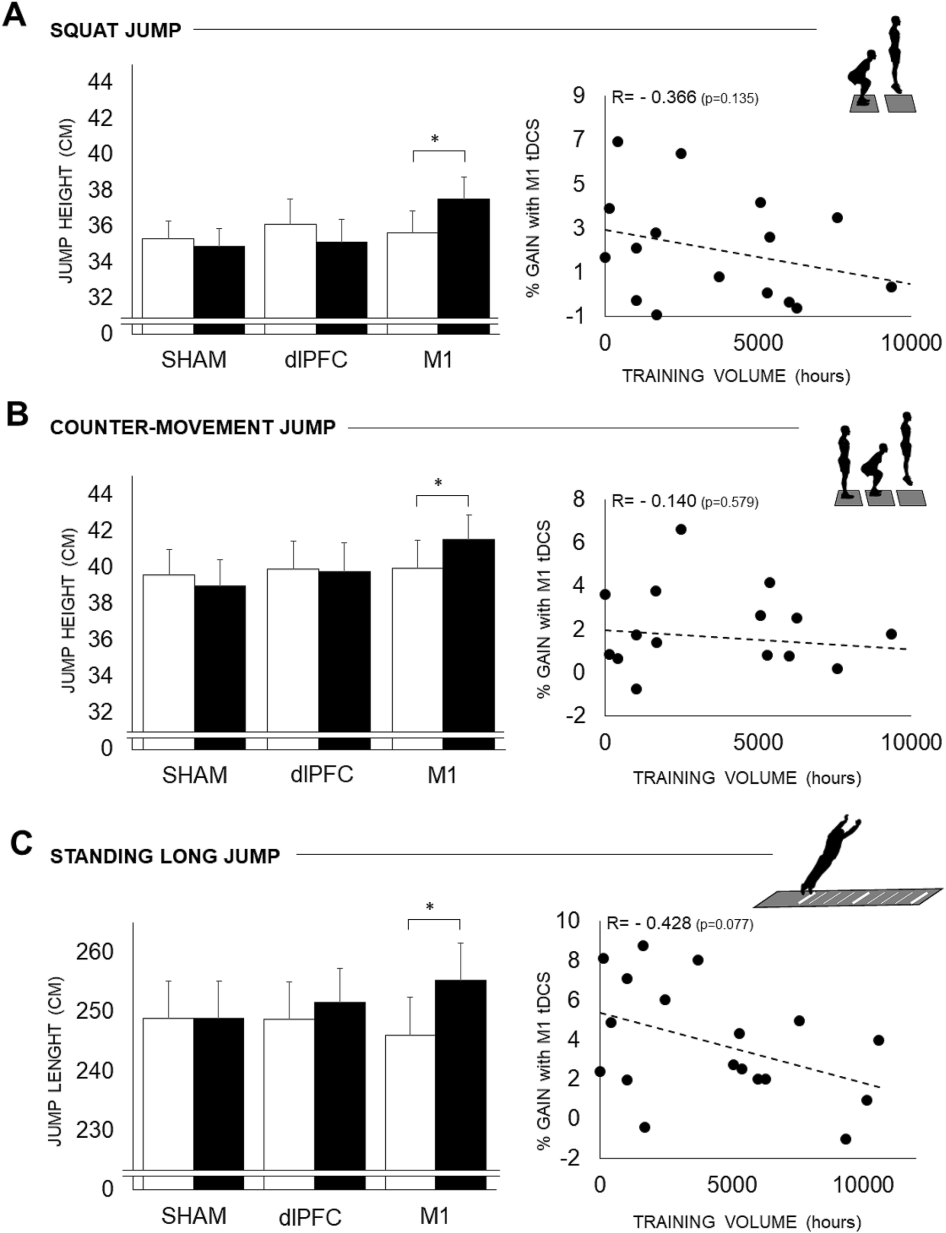
Jump performance before and after the three tDCS sessions. Data are mean ± SEM. Jump performances are depicted in cm for the vertical jumps, i.e., the squat jump (**A**) and the countermovement jump (**B**), and for the horizontal jump, i.e., the standing long jump (**C**). The left graphs display the performance before (PRE: white bars) and after (POST: black bars) each condition (SHAM: control condition; dlPFC: tDCS applied over the left dlPFC; M1: tDCS applied over the leg right primary M1). *pre-post statistical difference. The right graphs display the relationships between the relative gains of performance following M1 tDCS (in %) plotted against the total training volume of the athletes, calculated with experience (in years) and training frequency (hours/week).

A significant main effect of factor *condition* was found on PRE-POST changes in CMJ height (F_2,34_ = 6.60, P = 0.0038, η^2^ = 0.186), SJ height (F_2,34_ = 11.71, P = 0.0001, η^2^ = 0.262), and SLJ distance (F_2,34_ = 16.05, P < 0.0001, η^2^ = 0.376). No significant difference was observed in the pre- and post-performances for the vertical and horizontal jumps after the SHAM condition or after dlPFC (Fig. 2A–C). In contrast, a significant pre-post increase was observed in M1 for SJ (+ 5.3 ± 2.3%, P = 0.0035), CMJ (+ 4.0 ± 2.2%, P = 0.0071), and SLJ (+ 9.3 ± 6.2%, P < 0.0001), with the largest increase observed for SLJ. No specific relationship was found between the experience of the participants and the gains observed with tDCS (r = − 0.311 on average) (Fig. 2A–C).

### Cognitive and psychiatric assessments

Due to the multiplicity of tests, the significance thresholds were determined as follows: p < 0.008 for QIDS-C16 and QIDS-SR16, p < 0.005 for the EEfRT, and p < 0.002 for the other tasks. There was no significant difference between the reported (QIDS-SR16) and clinically assessed (QIDS-C16) depressive symptomatology before and after the participating in the protocol (Table 1). Examination of the BART test (decision-making behaviors) scores in comparison to the BIS-10 scale (BIS-10 total score, cognitive-, motor- and non-planning-impulsivity) and the MCQ did not reveal that tDCS had any significant main effect nor interaction on these variables (Table 2). The Stroop task, represented by the interference effect corresponding to the difference between the interference time and the denomination time, as well as the Go/No-go test score did not reveal any statistically significant difference. The main variable assessed for each condition of the EEfRT, that is the percentage of “hard” task choices, was analyzed first in aggregate and then in terms of the probability of retribution and the reward magnitude (Table 3). None of these analyses revealed a significant effect of tDCS, regardless of the type of stimulation that was used.

**Table 1:** Effects of tDCS on depressive symptomatology.

	Inclusion	Post protocol	p
QIDS-SR16	3.17 ± 2.01	3.17 ± 3.03	0.428
QIDS-C16	2.50 ± 1.76	2.11 ± 2.25	0.572

Data are mean ± SD. The results are obtained at the inclusion and at the end of the protocol.

*QIDS-SR16* 16-item Self-Report version of the Quick Inventory of Depressive Symptomatology, *QIDS-C16* 16-item Clinician version of the Quick Inventory of Depressive Symptomatology.

**Table 2:** Effects of tDCS on different aspects of impulsivity.

Condition	SHAM			dlPFC			M1		
	PRE	POST	p	PRE	POST	p	PRE	POST	p
**Risk taking (BART)**									
**Average adjusted pumps**									
Total	42.53 ± 16.46	45.83 ± 12.26	**0.095**	42.98 ± 10.87	42.87 ± 7.10	**0.962**	39.60 ± 15.51	46.52 ± 15.26	**0.027**
First 10	38.06 ± 17.97	43.50 ± 13.85	**0.229**	38.28 ± 13.11	40.33 ± 7.51	**0.709**	36.44 ± 17.48	38.83 ± 15.41	**0.058**
Middle 10	44.89 ± 19.38	47.20 ± 13.83	**0.385**	45.31 ± 14.15	44.82 ± 8.66	**0.872**	39.28 ± 14.36	46.59 ± 13.31	**0.028**
Last 10	47.71 ± 18.03	46.79 ± 11.92	**0.794**	46.06 ± 9.23	44.30 ± 9.09	**0.567**	39.64 ± 18.95	51.10 ± 19.04	**0.081**
**Response inhibition**									
Go/NoGo task	3.00 ± 0.00	2.83 ± 0.38	**0.250**	2.61 ± 0.50	2.89 ± 0.32	**0.125**	2.83 ± 0.38	3.00 ± 0.00	**0.250**
Stroop (interference effect)	25.13 ± 6.68	21.97 ± 9.03	**0.003**	29.59 ± 8.10	25.41 ± 9.83	**0.029**	28.27 ± 8.84	27.37 ± 11.24	**0.545**
**Delay discounting (MCQ)**									
*k* values	0.0136 ± 0.016	0.014 ± 0.02	**0.199**	0.0164 ± 0.02	0.0203 ± 0.03	**0.577**	0.0142 ± 0.01	0.0134 ± 0.01	**0.469**
**Self-assessment (BIS-10)**									
Global impulsivity	51.61 ± 11.92	50.83 ± 12.70	**0.413**	52.11 ± 12.90	52.56 ± 13.51	**0.240**	52.11 ± 13.38	51.94 ± 12.90	**0.854**
Motor-impulsivity	16.33 ± 7.90	15.72 ± 7.65	**0.238**	16.78 ± 7.64	16.22 ± 7.74	**0.652**	15.78 ± 7.95	15.17 ± 7.60	**0.301**
Non-planning-impulsivity	18.94 ± 5.16	18.33 ± 5.52	**0.276**	18.28 ± 5.91	19.50 ± 6.18	**0.033**	19.00 ± 5.42	19.33 ± 5.74	**0.579**
Cognitive-impulsivity	16.33 ± 2.68	16.78 ± 3.28	**0.386**	17.06 ± 3.06	16.83 ± 3.43	**0.594**	17.33 ± 3.58	17.44 ± 3.13	**0.821**

Data are mean ± SD. The results are obtained before (PRE) and after (POST) each condition (SHAM: control condition; dlPFC: tDCS applied over the left dorsolateral prefrontal cortex; M1: tDCS applied over the right primary motor cortex).

*BART* Balloon Analog Risk Task, *MCQ* Monetary Choice Questionnaire, *BIS-10* Barratt Impulsiveness Scale-10.

**Table 3:** Effects of tDCS on motivation during the Effort Expenditure for the Reward Task (EEfRT).

Condition	SHAM			dlPFC			M1		
	PRE	POST	p	PRE	POST	p	PRE	POST	p
**Proportion hard task choices (%)**									
Total trials	41.72 ± 19.35	42.01 ± 20.09	**0.632**	40.18 ± 19.42	40.02 ± 21.01	**0.963**	43.80 ± 26.00	40.04 ± 20.64	**0.927**
**Probability of retribution**									
12%	14.51 ± 25.23	12.81 ± 24.46	**0.427**	13.82 ± 24.26	10.47 ± 24.82	**0.207**	18.30 ± 33.58	12.31 ± 26.25	**0.313**
50%	39.55 ± 25.95	41.40 ± 29.12	**0.565**	38.36 ± 28.82	39.54 ± 30.24	**0.794**	41.97 ± 31.86	35.82 ± 26.84	**0.782**
88%	69.72 ± 17.70	70.56 ± 18.74	**0.693**	67.71 ± 11.98	68.76 ± 14.08	**0.739**	70.47 ± 16.96	70.94 ± 17.06	**0.426**
**Reward magnitude**									
< 1.96$	15.84 ± 24.88	13.30 ± 24.34	**0.244**	13.33 ± 22.36	12.29 ± 23.93	**0.530**	19.00 ± 32.69	15.49 ± 25.38	**0.244**
1.96 < $ < 2.77	35.36 ± 23.94	37.06 ± 24.18	**0.441**	33.75 ± 24.32	33.88 ± 26.30	**0.975**	35.68 ± 30.03	31.51 ± 22.53	**0.441**
2.77 < $ < 3.96	51.87 ± 19.31	53.24 ± 21.34	**0.603**	51.37 ± 17.91	52.09 ± 20.56	**0.806**	55.49 ± 24.13	52.02 ± 21.64	**0.603**
> 3.96$	62.57 ± 21.18	62.54 ± 22.16	**0.992**	59.20 ± 22.48	59.45 ± 19.30	**0.946**	62.56 ± 23.21	58.96 ± 21.63	**0.992**

Data are mean ± SD. The proportion of hard task choices is assessed across all choices and according to the probability of reward (12, 50 or 88%) and its magnitude. The results are obtained before (PRE) and after (POST) each condition (SHAM: control condition; dlPFC: tDCS applied over the left dorsolateral prefrontal cortex; M1: tDCS applied over the right primary motor cortex).

### Plantar flexors’ neurophysiological data

**Figure 3:**
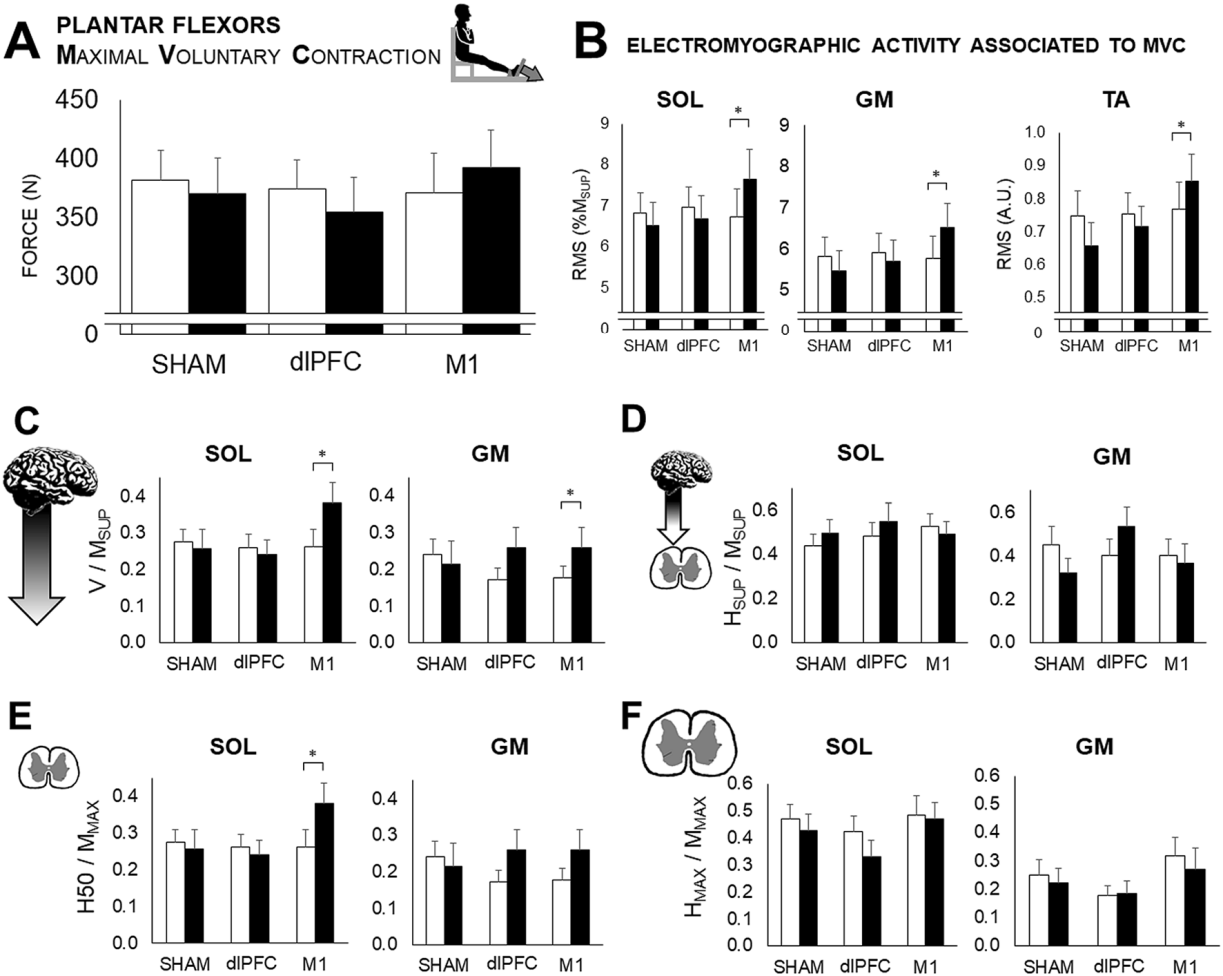
Neuromuscular assessment. Data are mean ± SEM. The data are displayed before (PRE: white bars) and after (POST: black bars) each condition (SHAM: control condition; dlPFC: tDCS applied over the left dlPFC; M1: tDCS applied over the leg right primary M1). Data for the soleus (SOL), gastrocnemius medialis (GM), and tibialis anterior (TA) are depicted. (**A**) Force associated with maximal voluntary contraction (MVC) of the plantar flexors. (**B)** MVC-associated EMG activity of SOL, GM, and TA. (**C**) supraspinal index, V-wave, associated to MVC. (**D**) Superimposed H-reflex (H_SUP_) to MVC. (**E)** Submaximal rest spinal excitability (H_50_). (**F**) Maximal rest H-reflex (H_MAX_). *pre-post statistical difference.

No significant effect of any of the tDCS conditions (F_2,34_ = 2.34, P = 0.0754, η^2^ = 0.086) was shown on the plantar flexors’ MVC (Fig. 3A). Nevertheless, a significant effect of factor condition has been found in myoelectrical activity associated to MVC, as expressed by RMS/M_SUP_ ratios (SOL: F_2,34_ = 11.88, P = 0.0001, η^2^ = 0.250; GM: F_2,34_ = 12.06, P = 0.0001, η^2^ = 0.249). In contrast to the dlPFC and SHAM conditions, a significant pre-post increase of SOL (P < 0.001), GM (P = 0.0016), but also TA (P < 0.001) RMS/M_SUP_ was found following M1 tDCS (Fig. 3B). This increased activity after M1 tDCS was accompanied by an increase of V/M_SUP_ in SOL (P < 0.001) and GM (P = 0.0011); no effect was observed on H_SUP_/M_SUP_. No statistical pre-post difference was observed in the SHAM and dlPFC conditions for V/M_SUP_ and H_SUP_/M_SUP_ (Fig. 3C,D).

A significant main effect of condition has been found on submaximal H-reflex (H_50_/M_MAX_) for SOL muscle (F_2,34_ = 9.65, P = 0.0005, η^2^ = 0.243). The ratio was significantly enhanced after M1 for SOL (P = 0.0002). No significant effect has been found in GM muscle (P = 0.0029), however this statistical result is barely above the corrected threshold [0.0022] according to Bonferroni’s correction(Fig. 3E). No statistical pre-post difference was found for H_MAX_/M_MAX_ for any of the tDCS sessions (Fig. 3F). In SOL and GM, no statistical difference was found on the muscle potentials for rest (M_atH50_, M_atHmax_, M_MAX_) and for the superimposed responses (M_atHsup_, M_SUP_).

### Fine motor skills

**Figure 4:**
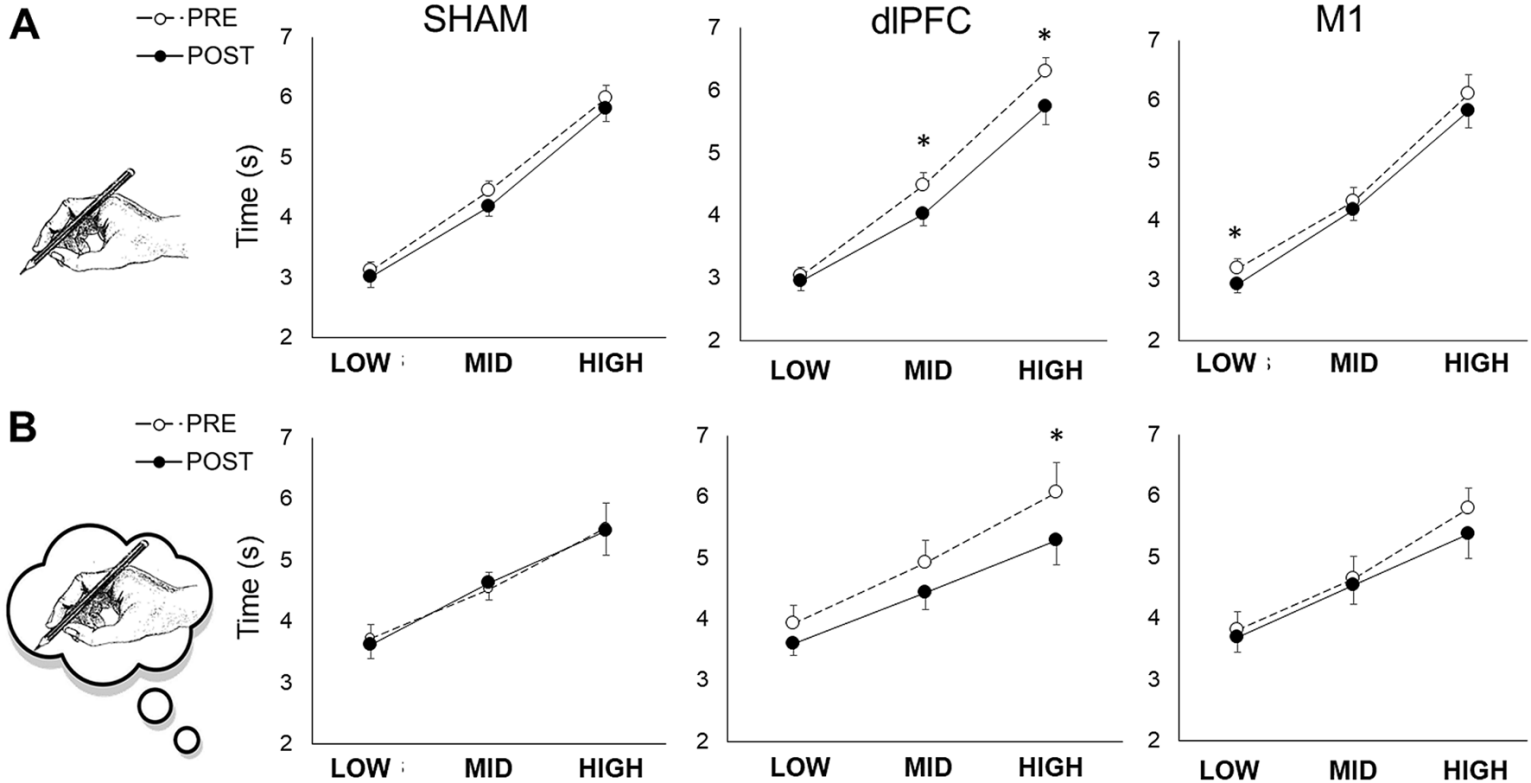
Actual and imagined pointing tasks. Data are mean ± SEM. The data are displayed before (PRE: white circles) and after (POST: black circles) for each condition (SHAM: control condition; dlPFC: tDCS applied over the left dlPFC; M1: tDCS applied over the leg right primary M1). (**A)** Time to perform actual pointing tasks in the three conditions for the three indexes of difficulty (ID): low, medium, and high difficulties. (**B**) Time to mentally perform the task using motor imagery in the three conditions for the three IDs. *statistical pre-post difference.

In all the conditions, no significant differences were observed between the imagined and actual pointing tasks. The time to perform each task was not affected by the SHAM condition (Fig. 4A,B). A significant pre-post decrease was observed for the dlPFC condition for actual pointing time at medium ID (P = 0.0010, d = 0.583) and high ID (P = 0.0014, d = 0.471), and at high ID for the imagined pointing time (P = 0.0010, d = 0.467). A significant pre-post decrease in the actual pointing time in the low ID (P = 0.0059, r = − 0.450) was found for the M1 condition (Fig. 4A).

## Discussion

The present study aimed to test the acute effect of several tDCS montages on the cognitive and motor performances of athletes practicing parkour. While no effect was found on the cognitive and psychiatric evaluations for any of the tDCS conditions, it appears that tDCS, applied over M1 with an extracephalic cathode, enhanced the participants’ jump performances, accompanied by changes at the supraspinal and spinal levels. The dlPFC tDCS mainly exhibited changes in the actual and imagined times needed to perform a hand pointing task.

Contrary to our expectations based on the literature, a 20-min offline stimulation of the left dlPFC or the right M1 was not sufficient to induce behavioral and cognitive changes in several domains: response inhibition, risk-taking, delay discounting, and motivation. Stimulation parameters, with key elements, such as current density or the electrodes’ position, are essential moderators that can explain the heterogeneity of the results in cognitive studies. Since the diversity of the rationale in the current literature is extremely wide, establishing consistent comparisons between the trials and their outcomes appears to be difficult (for a review see Teti Mayer et al.^30^). Another possible explanation is that accomplishing a cognitive task involves several cognitive regions; thus, modulating one region of the entire network is less likely to affect cognitive performances when compared to motor changes.

Regarding motor changes, only a few studies have referred to the effect of tDCS on jump performance, and they have reported contradictory results. The study by Lattari et al.^12^ also found an increase in CMJ jump following 20 min of offline tDCS at 2 mA, with a different montage (anode on Cz and cathode on Fp2). In contrast, Mesquita et al.^14^ reported no effect following offline tDCS, but they used a lower intensity (1.5 mA) and a shorter duration (15 min) with a different montage (anode: bilateral C3 and C4; cathode: ipsilateral shoulder). Romero-Arenas et al.^13^ stimulated the dlPFC offline at 1.5 mA for 15 min (anode: F3, cathode: Fp2); they did not report any effect, which is similar to the present study’s results for the dlPFC session. Here, the effect of tDCS on jump performances was observed with anodal tDCS on M1, particularly with an extracephalic cathode. Importantly, in addition to repelling the negative effect of the cathode, the current densities are up to 4.4-times higher with an extracephalic tDCS montage, which could possibly increase the number of responding participants^31^.

Notwithstanding, dlPFC stimulation appeared to qualitatively alter the motor strategies of fine motor skills, as evidenced by a change in the relationship between pointing time and task difficulty (Fitt’s Law). While a global shift in the relationship is generally ascribed to a modulation of motor command noise that interferes with the variability of the final position of the pointing movement^32^, an apparent change in its slope indicates a change in motor strategies at earlier stages of motor planning. This was emphasized by a similar behavior observed on the highest ID during the imagined pointing time test. MI is thought to represent all motor preparation processes without motor output and concomitant sensory feedbacks^33^, by sharing similar brain activations with actual movement^34,35^. Therefore, such a modulation of Fitts’ Law regression during MI following dlPFC tDCS indicated changes in motor planning strategies, while changes in actual movement duration reflects all the aspects of the movement, including motor execution^32^. Therefore, measuring both MI and actual movement time could represent an interesting model to distinguish among the several levels^32^. This modulation of simulated movement time may involve optimization of the internal forward model, thus predicting the future state of the system^36^. This is in accordance with the results reported in a previous study, which found an improvement in mental hand rotation tasks following offline prefrontal tDCS^37^.

To summarize, it appears that tDCS does not lead to a global arousal of brain motor function; rather, the montage appears to be crucial for the type of effects desired^38^, as evidenced here by the task-specific effect of tDCS applied either over M1 or dlPFC.

While cognitive function can be largely modulated in frail populations, tDCS might be an insufficient stimulus to alter a healthy, optimally performing brain. In addition to the nature of the population being tested (patients, healthy), a large inter-individual variability has also been acknowledged to explain the discrepancies in the tDCS effect in the literature^39^ based on structural factors, such as neuroanatomy or brain circuitry^40^. In terms of the sport performance-related effects, participants’ expertise has also been suggested as one of the main requirements^12^. Lattari et al.^12^ recruited well-trained participants (weightlifters); other studies recruited recreational participants^13^ or non-power specialists^14^. The present study included parkour practitioners with a long-term experience in jumping^41^. This population may present pre-cabled motor areas for the tested performances, as tDCS reinforced existing networks by synaptic long-term potentiation^38^. As evidence, the largest effect of M1 tDCS was observed on SLJ (+ 9%), which is a technique that is highly specific to parkour in comparison to vertical jumps^17^. Moreover, this technique showed a nearly significant (P = 0.07) correlation between the level of expertise, quantified here as the total training volume, and the gains observed with M1 tDCS (Fig. 2C). The positive effect of tDCS seemed to decrease with experience, possibly raising a ceiling effect. Nonetheless, overall any correlation appeared significant within such a group of trained individuals, the level of expertise did not seem to be a predictive factor of the tDCS effect.

It has been suggested that an increase in the cortical motor drive is one of the main factors to explain the effect of tDCS on power performance^12^, although conflicting results can be found in the literature^27^. The present study’s result showed a lack of effect on isometric MVC in the participants’ calve muscles, similar to previous works, and no effect was found on the knee extensors’ maximal force using similar stimulation parameters^42^. The lack of representativeness of isometric force to functional tasks, such as jumps, might then be opposed. However, surprisingly, an increase in leg muscle EMG activity could still be observed on all the tested muscles, including SOL, GM, and TA. Therefore, it is possible that the scope of tDCS over the M1 area was not muscle-specific and the proximity of the dorsi- and plantar-flexor cortical motor representations led to a concomitant increase in the activation of both muscle groups. Since they act as antagonists, this could potentially hide modulation on the maximal force of one muscle or the other. However, such a global arousal of the activation of leg muscles may be beneficial for a more-complex and whole-body activity, such as jumps.

An increase in EMG activity following M1 tDCS indicates an effect on the motor command processes. Numerous studies have shown an enhancement of M1 corticospinal excitability following tDCS, even in small brain regions such as the leg motor cortex^28^. In the present study, an increase in V/M_SUP_ was observed, which is usually used as an indirect index of the cortico-motoneuronal neural drive^43^. This argues in favor of the optimization of neural recruitment, as previously evidenced on motor-evoked potential^28,44,45^. However, since these indexes involve the entire corticospinal tract, a potential contribution of lower nervous levels cannot be precluded. Indeed, tDCS has been shown to enhance motor unit synchronization^9,10^, which is largely influenced by spinal circuitry^46^. In the present study, an increase in submaximal spinal excitability (H_50_/M_SUP_) was observed. Notwithstanding, a lack of maximal H-reflex modulation (H_MAX_ or H_SUP_) indicated that tDCS did not lead to a global arousal of spinal excitability; rather, it targeted more sensitive structures. More precisely, a partial removal of spinal inhibitions without changes in maximal H-reflex was already found following 20 min of offline M1 tDCS^29,47^. Roche et al. also found a positive effect of M1-tDCS to modulate lumbar propriospinal system excitability, a sub-cortical interneuronal circuitry that regulates the motor command of the lower limb^48,49^. In general, spinal inhibitory interneurons are more sensitive to sub-threshold cortical outputs than alpha motoneurons^50^.

The specificity of the population tested could also explain this effect; parkour athletes present a particularly low spinal excitability^18,51^, which makes them prone to spinal-induced tDCS plasticity. The electrode in our M1-tDCS condition, i.e., an extracephalic cathode, was also optimal to involve a larger part of the neural pathway, including the medullar levels, than the usual montage with the cathode placed on the suborbital frontal region.

The lack of effect of dlPFC tDCS on the leg neuromuscular parameters indicates that the connectivity of the dlPFC and M1 areas could not be strong enough to modulate the motor command. Nonetheless, if dlPFC is mainly attributed to decision processes and working memory, our results for the actual and imagined Fitt’s pointing task still emphasized an intimate link between dlPFC and the motor planning of complex actions^52^. Using functional magnetic resonance, it was found that dlPFC was activated when the task requires an individual to mentally prepare a sequential action based on short-term memory^53^. Therefore, it appears that dlPFC could play a role in motor planning for both imagined and actual pointing tasks, since this area is activated during both MI and actual contraction^54^. The relationship between dlPFC and the basal ganglia^52^, which regulates planning of complex motor sequences, could also explain the effect of tDCS on the motor strategies of an accurate pointing task. In contrast, the jumping tasks did not benefit from this stimulation, probably because the particular experience of the participants did not leave any room for a potential optimization in motor planning of such a usual task.

This study has some limitations. First, the lack of retribution/financial reward of the cognitive tasks could have limited the motivation and risk-taking behavior of our participants. Second, despite no session-order effect has been statistically evidenced, a potential learning effect across the experimental sessions, even small, could have hidden the effects induced by tDCS, even though the tasks and tests were randomized. Third, due to the duration of each experimental session and the multiplicity of the tests performed, cognitive fatigue and/or boredom could have interfered with the psychometric evaluation. Therefore, all these factors underline the difficulty of specifically determining the effects of tDCS on impulsivity and motivation, which leads us to consider our results with caution. Moreover, given the duration required to measure all tested variables POST-tDCS treatment, the effect of brain stimulation could have partially vanished when cognitive measures were performed. Even though the effect of anodal tDCS on cortical excitability were shown to be still important 60 min after the end of the stimulation^55^, it could be argued that the optimal effects are rather observed directly after the tDCS application. Regarding cognitive functions, the online effect of tDCS could also be relevant to assess, by performing some of the tests during the stimulation.It should be pointed out that the lack of effect of M1 tDCS on hand pointing tasks in the present study does not rule out a possible effect of M1 tDCS on speed accuracy trade-off. Indeed, in the present study the localization of anode electrode has been optimized for leg performance. Therefore, the stimulation of leg representation in the motor cortex of the right hemisphere is definitely not the optimal target to enhance right hand performance. A more optimized setup to address the effect of tDCS on speed-accuracy tradeoff management is necessary to deepen the analysis of this parameter.

It was suggested that a better performance following tDCS could be the result of the low spatial resolution of the stimulation, possibly affecting many areas involved in motor control^56^. It should be noted that the type of tDCS used here was not focal, resulting in a stimulation of numerous areas under the electrodes. However, this could be an advantage when aiming to improve a motor performance that involves a complex and large brain network.

To conclude, in an absence of any cognitive emulation, stimulation of dlPFC was likely to affect tasks requiring fine motor control, while M1 applied over the leg motor area with an extracephalic cathode was likely to enhance power performances through the effective solicitation of a large part of the corticospinal pathway. However, generalizing this assumption to other performances and populations may lead to an over-simplification of the motor system, particularly because tDCS effects can depend upon a wide variety of motor stimulated areas and stimulation parameters. It was suggested that brain stimulation would have a more pronounced effect on the functional connectivity of pre-existing neural circuits, increasing well-known performances rather than unknown tasks, which implies motor learning and the creation of new connections^57^. Finally, as suggested by Roche et al. in 2009^29^, the present study also emphasized the importance of investigating a large part of the neural pathway when considering the neural correlates of tDCS motor performance enhancement, including spinal network connectivity.

## Methods

The study’s methods and materials will be described concisely, since the full rationale has been previously published in a specific “study protocol” article^19^.

### Participants

Eighteen healthy young males, all over 18 years old, (age: 22.6 ± 5.7 years old; height: 180 ± 5.7 cm, weight: 74.5 ± 7.8 kg) gave written informed consent to participate in the study. The participants were all right-handed and they did not report neurological or physical disorders, or psychiatric or addictive comorbidities. Participants’ handedness has been evaluated by the Edimburgh Inventory^58^. All the participants indicated their sport involvement in years and their mean training frequency in hours/weeks, from which the total number of training hours (experience) was calculated. Since we wanted to address the effect of experience (training volume) on tDCS effect, participants of various training volume have been recruited (mean: 4343.4 ± 3508.1 h, minimum: 156, maximum: 10,608).

The research protocol, registered on ClinicalTrials.org (NCT03937115, first registration: 03/05/2019), was approved by the Institutional Review Board (Comité de Protection de Personnes (CPP)-Est-4, number 18/47), and the study was conducted in accordance with the last version of the Declaration of Helsinki.

### Experimental design

This study was a double-blind, randomized, sham-controlled trial to determine the effects of tDCS applied over the right M1 and the left dlPFC on motor and cognitive performances. To ensure the blinding of the type of tDCS applied toward participants, they were told that any of the three experimental conditions could be a placebo session, independently of the electrode placement. Regarding blinding of the experimenters, it should be mentioned that the operator that applied the tDCS was not the same as the one who performed pre-post tests. An overview of the setting of the present study is presented in Fig. 1 A–E.

Each participant made three visits to the laboratory. The first visit included an interview with a trained psychiatrist to ensure that the participant met the inclusion criteria. Then, three different tDCS settings were randomly tested in different experimental sessions separated by at least 48 h, always organized in the same way (Fig. 1A). Cognitive, motor, and neuromuscular assessments were performed before and after each of the tDCS conditions.

### Transcranial direct current stimulation (tDCS)

Direct current was applied through the scalp using a neurostimulator system (StarStim, Neuroelectrics, Barcelona, Spain) with a SHAM condition and double-blind mode. The anode and cathode were two saline-soaked sponge electrodes (Sponstim, 25 cm^2^) placed on a neoprene head cap. Two different montages were used in this procedure: (1) the anode placed over the right M1 (corresponding to FC2 according to the international 10–20 EEG system) and the cathode placed over the left shoulder (M1 condition); (2) the anode placed over the left dlPFC and the cathode placed over the right supraorbital region, corresponding to F3 and AF8 according to the international 10–20 EEG system (dlPFC condition). These montages were chosen to optimize the stimulation of each zone. Indeed, while for the dlPFC it is recommended to place the cathode on a supraorbital area, for M1 it is recommended, especially for physical performance purposes, to use an extracephallic cathode^41^. The third condition consisted of a placebo stimulation (SHAM condition) with the same montage as the dlPFC condition. In both active sessions, the current was progressively increased during the first 30 s, maintained at 2 mA for 20 min, and then progressively decreased for 30 s. In the SHAM session, stimulation was turned off after 30 s, allowing the participant to feel the initial itching sensations associated with tDCS, thus representing a valuable placebo condition^59^. Furthermore, the participants were asked after each session to report whether they felt the stimulation was active or placebo. The error rate was 44.44% and the majority of subjects (55.6%) thought they had received active stimulation during the placebo session. No difference has been reported in adverse events experienced (tingling, itching, etc.) between the placebo and the active sessions.

### Cognitive and psychometric assessments

The tDCS effects on different aspects of impulsivity were assessed before and after each session by a trained psychiatrist. Global impulsivity and its three sub-dimensions (motor impulsivity, cognitive impulsivity, and lack of planning) were measured using the French version of the Barratt Impulsiveness Scale (BIS-10)^60^. Risk taking was assessed with the Balloon Analog Risk Task (BART)^23^. Attention and inhibitory control were estimated using the Go/No-go^24^ and Stroop tasks^25^, and delay discounting was assessed by the 27-item Monetary Choice Questionnaire (MCQ)^26^. The tDCS effects on motivation were assessed on a computer interface with the Effort Expenditure for Reward Task (EEfRT)^22^. Finally, before the first experimental session and after the last session, depressive symptomatology was assessed using the 16-item Clinician and Self-Report versions of the Quick Inventory of Depressive Symptomatology (QIDS-C16 and QIDS-SR16)^61^.

### Fine motor skills: speed-accuracy tradeoff

In a sitting position, the participants had to use a pencil (right hand: dominant hand) to alternately point at two targets as accurately and as quickly as possible. The index of difficulty (ID) was set according to the target widths and the distance between the targets (Fig. 1). Two trials were performed per the ID (total of six trials); in each trial, the participants pointed 10 times between the targets. The average of these two trials was taken for analysis. Similarly, the participants had to imagine themselves performing these six trials. They were instructed to feel themselves pointing between the targets (kinesthetic motor imagery [MI]) as they if they actually doing it.

### Motor performance assessments

After a brief warm up, the horizontal jump performances were characterized by the maximal metered performance in the standing long jump (SLJ). Additionally, two types of vertical jumps were performed on a force plate (Kistler, Winterthour, Switzerland): the squat jump (SJ) and the counter movement jumps (CMJ) (Fig. 1). Maximal height and distance were taken into account among the several trials. The maximal force of the plantar flexors was also assessed to account for neuromuscular plasticity by recording the isometric maximal voluntary contraction (MVC) on a pedal equipped with a constraint gauge (PCE Instruments, Strasbourg, France).

### Neuromuscular assessments

Neuromuscular assessments were performed while the participants were seated in a comfortable chair in a relaxed position (Fig. 1). Electromyographic (EMG) activity was recorded during the tests from four muscles of the left leg (soleus [SOL], gastrocnemius medial [GM], tibialis anterior [TA], vastus lateralis [VL]). EMG signals were recorded with wireless Trigno sensors (Delsys, Natick, MA, USA) and digitized online (sampling frequency: 2 kHz) with Labchart software (LabChart 8, ADInstruments, Sydney, Australia).

Neuromuscular function was assessed by recording the motor potentials of SOL and GM evoked by stimulating the posterior tibial nerve. At rest, three potentials were recorded: maximal (H_MAX_), submaximal H-reflex (H_50_, corresponding to 50% of H_MAX_), and maximal muscle compound action potential (M_MAX_). At the muscle level, M_MAX_ characterizes the direct activation of the muscle at the neuromuscular junction; at the spinal level, H_50_ and H_MAX_ reflect the Ia-to-alpha motoneuronal transmission. Maximal H-reflex and M-wave were also superimposed to MVC, then noted as H_SUP_ and M_SUP_, respectively. It can be noted that M_SUP_ is followed by a V-wave, which is used as an index of the supra-spinal descending neural drive^43^. All the responses were normalized to the maximal M-wave evoked in the same condition; therefore, H_MAX_/M_MAX_, H_50_/M_MAX_, H_SUP_/M_SUP_, and V/M_SUP_ were considered.

### Statistical analysis

One-way repeated-measures analysis of variance (ANOVA) was performed on delta (PRE-POST) with factor *condition* (dlPFC, SHAM, M1), or a Friedman test if normality was not verified. The Shapiro test assessed the normality of each variable. If the normality is verified on the 3 conditions for a variable, then a Mauchley test was performed to evaluate the sphericity (equality of variances). If the sphericity was not verified, then a Huynh–Feldt adjustment replaces the p-value.

Paired Student t tests or Wilcoxon's signed rank tests were also performed to compare the results before (PRE) and after (POST) the tDCS sessions. Bonferroni corrections were used to correct for type I errors due to multiple testing; P < 0.0167 was considered statistically significant for the primary endpoints (CMJ, SJ, SLJ). For the secondary endpoints (motor, cognition, EEfRT, and pointing), type I errors were fixed depending on the number of parameters that were used and tested. Pearson or Spearman correlations were performed to account for possible correlations between the variables. P-values should be interpreted with caution because of the small sample size and the multiplicity of testing. Regarding the effect sizes, for ANOVAs eta-squared were calculated, Cohen’s d for student analysis and r effect sizes for Wilcoxon tests. All statistical analyses were performed using SAS 9.4 software (Tables S1, S2).

## Supplementary Information

Supplementary Table S1.

Supplementary Table S2.

## Abbreviations

BART: Balloon Analog Risk Task (BART)
BIS-10: Barratt Impulsiveness Scale
CMJ: Counter movement jump
dlPFC: Dorsolateral prefrontal cortex
EEfRT: Effort Expenditure for Rewards Task
EMG: Electromyography
GM: Gastrocnemius medialis muscle
M1: Primary motor cortex
MCQ: Monetary Choice Questionnaire
MI: Motor imagery
MVC: Maximal voluntary contraction
QIDS: Quick Inventory of Depressive Symptomatology
RMS: Root mean square
SJ: Squat jump
SLJ: Standing long jump
SOL: Soleus muscle
TA: Tibialis anterior muscle
tDCS: Transcranial direct current stimulation
VL: Vastus lateralis muscle
