# Supplementary material for: Effect of transcranial direct current stimulation on the psychomotor, cognitive, and motor performances of power athletes
Source: Sci Rep. 2021 May 6;11:9731. doi: 10.1038/s41598-021-89159-7 (PMC8102586; doi:10.1038/s41598-021-89159-7)
Supplement: Supplementary file 1 — Supplementary Table S1. [file 41598_2021_89159_MOESM1_ESM.docx]

| Variable (Δ post-pre) | Sham | dlPFC | M1 | dF | F | P-value |
| --- | --- | --- | --- | --- | --- | --- |
| **Motor** |  |  |  |  |  |  |
| CMJ Height | -0.60±1.60 | -0.14±2.24 | 1.59±2.20 | 2,34 | 6.60 | 0.0038* |
| SLJ lenght | -0.06±3.96 | 2.80±5.24 | 9.33±6.20 | 2,34 | 16.05 | <0.0001* |
| SJ Height | -0.44±1.36 | -1.00±2.55 | 1.88±2.36 | 2,34 | 11.71 | 0.0001* |
| MVC | -11.30±46.43 | -19.13±68.00 | 21.50±61.07 | 2,34 | 3.13 | 0.0754 |
| SOL activation | -0.27±0.86 | -0.30±1.18 | 0.89±0.90 | 2,34 | 11.88 | 0.0001* |
| GM activation | -0.21±0.78 | -0.34±0.99 | 0.76±0.86 | 2,34 | 12.06 | 0.0001* |
| VL activation | 0.04±0.32 | 0.02±0.14 | -0.04±0.17 | 2,34 | 0.42 | 0.6582 |
| SOL H_50_/M_max_ | -0.02±0.14 | -0.02±0.13 | 0.13±0.12 | 2,34 | 9.65 | 0.0005* |
| SOL H_sup_/M_sup__ | 0.06±0.21 | 0.06±0.27 | -0.02±0.16 | 2,34 | 0.9 | 0.4172 |
| SOL V/M_sup__ | 0.01±0.19 | 0.03±0.11 | 0.14±0.10 | 2,34 | 4.30 | 0.0346 |
|  |  |  |  |  |  |  |
| **Cognitive** |  |  |  |  |  |  |
| BART (Av Adj Pump)  Middle 10 | 2.31±11.00 | -0.49±12.65 | 7.31±12.95 | 2.34 | 1.96 | 0.1561 |
| Stroop (interference effect) | -3.16±3.90 | -4.18±7.42 | -0.90±6.18 | 2,34 | 1.33 | 0.2769 |
| Cognitive impulsivity | 0.44±2.12 | -0.22±1.73 | 0.11±2.05 | 2.34 | 0.42 | 0.6595 |
| Non-planning impulsivity | -0.61±2.30 | 1.22±2.24 | 0.33±2.50 | 2.34 | 3.32 | 0.0483 |

Table S1: Results of ANOVA. Data are Mean±SD. Δ post-pre are obtained by the difference between the results before and after stimulation (SHAM: control condition; dlPFC: tDCS applied over the left dorsolateral prefrontal cortex; M1: tDCS applied over the right primary motor cortex). *dF:* Degree of Freedom; *F:* statistic; *CMJ:* Counter Movement Jump; *SLJ:* Standing Long Jump; *SJ:* Squat Jump; *MVC*: Maximum Voluntary Contraction; *SOL*: Soleus; *GM:* Gastrocnemius Medialis; *VL*: Vastus Lateralis; *H_50_*: submaximal H-reflex; *M_max_:* maximal muscle compound action potential; *H_sup_*: Maximal H-reflex superimposed to MVC; *M_su_*_p_: M-wave superimposed to MVC; *V:* V-wave*; BART*: Balloon Analog Risk Task; *Av Adj Pump:* average adjusted pump; *significant result (Bonferroni corrections were used to correct for type I errors due to multiple testing; only P<0.0167 were then considered statistically significant).
