# Supplementary material for: Effect of transcranial direct current stimulation on the psychomotor, cognitive, and motor performances of power athletes
Source: Sci Rep. 2021 May 6;11:9731. doi: 10.1038/s41598-021-89159-7 (PMC8102586; doi:10.1038/s41598-021-89159-7)
Supplement: Supplementary file 2 — Supplementary Table S2. [file 41598_2021_89159_MOESM2_ESM.docx]

| Variable (Δ pre-post) | Sham | dlPFC | M1 | dF | T | P-value |
| --- | --- | --- | --- | --- | --- | --- |
| **Motor** |  |  |  |  |  |  |
| TA activation | -0.04±0.25 | -0.09±0.32 | 0.08±0.07 | 2 | 6.96 | 0.0308 |
| SOL M_atH50_/M_max_ | 0.04±0.13 | 0.02±0.05 | 0.02±0.18 | 2 | 0.34 | 0.8425 |
| SOL H_max_/M_max_ | -0.04±0.12 | -0.09±0.20 | -0.04±0.17 | 2 | 1.78 | 0.4111 |
| SOL M_atHmax_/M_max_ | 0.04±0.14 | 0.09±0.19 | 0.08±0.18 | 2 | 0.87 | 0.6462 |
| SOL M_max_ | -0.11±0.31 | -0.22±1.16 | -0.08±0.44 | 2 | 3.11 | 0.2111 |
| SOL M_atHsup_/M_sup_ | -0.03±0.25 | -0.02±0.11 | 0.05±0.25 | 2 | 0.44 | 0.8007 |
| SOL M_sup_ | 0.15±0.99 | -0.23±0.97 | 0.08±0.98 | 2 | 1.33 | 0.5134 |
| GM H_50_/M_max_ | -0.03±0.17 | 0.03±0.16 | 0.09±0.11 | 2 | 6.33 | 0.0421 |
| GM M_atH50_/M_max_ | 0.27±0.53 | 0.06±0.36 | 0.10±0.33 | 2 | 0.25 | 0.8809 |
| GM H_max_/M_max_ | -0.03±0.21 | 0.01±0.13 | -0.04±0.19 | 2 | 2.11 | 0.3480 |
| GM M_atHmax_/M_max_ | 0.27±0.52 | 0.13±0.45 | 0.09±0.26 | 2 | 3.44 | 0.1787 |
| GM M_max_ | 0.04±0.51 | 0.05±0.82 | -0.16±0.97 | 2 | 0.11 | 0.9460 |
| GM H_sup_/M_sup_ | -0.13±0.30 | 0.13±0.37 | 0.01±0.37 | 2 | 1.77 | 0.4118 |
| GM M_atHsup_/M_sup_ | -0.12±0.41 | 0.01±0.30 | -0.01±0.19 | 2 | 0.78 | 0.6778 |
| GM V/M_sup_ | 0.08±0.73 | 0.13±0.37 | 0.14±0.17 | 2 | 3.11 | 0.2111 |
| GM M_sup_ | 0.76±1.45 | -0.17±1.32 | -0.10±1.04 | 2 | 6.78 | 0.0337 |
|  |  |  |  |  |  |  |
| **Cognitive** |  |  |  |  |  |  |
| BART (Av Adj Pump) | 3.30±7.93 | -0.11±9.97 | 6.93±12.28 | 2 | 6.33 | 0.0421 |
| Go/NoGo task | -0.17±0.38 | 0.28±0.57 | 0.17±0.38 | 2 | 9.45 | 0.0089 |
| MCQ (k values) | 0.00±0.01 | 0.00±0.01 | -0.01±0.00 | 2 | 6.25 | 0.0438 |
| Global impulsivity | -0.78±3.93 | 0.44±4.05 | -0.17±3.79 | 2 | 4.49 | 0.1062 |
| Motor impulsivity | -0.61±2.12 | -0.56±2.79 | -0.61±2.43 | 2 | 0.57 | 0.7531 |
| Hard task choice (total) | 0.29±8.01 | -0.16±9.71 | -3.76±19.40 | 2 | 0.37 | 0.83 |
| Hard task choice (12%) | -1.70±8.89 | -3.35±9.23 | -5.99±19.28 | 2 | 0.31 | 0.856 |
| Hard task choice (50%) | 1.84±13.30 | 1.18±18.85 | -6.16±24.28 | 2 | 1.06 | 0.589 |
| Hard task choice (88%) | 0.85±8.94 | 1.05±13.15 | 0.47±19.68 | 2 | 0.54 | 0.765 |
| Hard task choice (<1.96$) | -2.54±8.92 | -1.04±6.84 | -3.51±21.85 | 2 | 0.14 | 0.933 |
| Hard task choice (1.96<$<2.77) | 1.70±9.14 | 0.13±17.36 | -4.17±28.83 | 2 | 0.22 | 0.898 |
| Hard task choice (2.77<$<3.96) | 1.37±10.99 | 0.72±12.30 | -3.47±18.89 | 2 | 0.00 | 1.00 |
| Hard task choice (>3.96$) | -0.03±11.69 | 0.25±15.27 | -3.59±21.61 | 2 | 0.09 | 0.956 |

Table S2 : Results of Test of Friedmann. Data are Mean±SD. Δ post-pre are obtained by the difference between the results before and after stimulation (SHAM: control condition; dlPFC: tDCS applied over the left dorsolateral prefrontal cortex; M1: tDCS applied over the right primary motor cortex). *dF:* Degree of Freedom; *T:* statistic; *SOL*: Soleus; *GM:* Gastrocnemius Medialis; *TA*: Tibialis Anterior; M_atH50_, M_atHmax_: muscle potentials for rest; *H_50_*: submaximal H-reflex; *M_max_:* maximal muscle compound action potential; *H_sup_*: Maximal H-reflex superimposed to MVC; *M_su_*_p_: M-wave superimposed to MVC; *V:* V-wave*; BART*: Balloon Analog Risk Task; *Av Adj Pump:* average adjusted pump; *MCQ:* Monetary Choice Questionnaire *significant result (Bonferroni corrections were used to correct for type I errors due to multiple testing; only P<0.0167 were then considered statistically significant).
